# Baseline and lifetime alcohol consumption and risk of skin cancer in the European Prospective Investigation into Cancer and Nutrition cohort (EPIC)

**DOI:** 10.1002/ijc.34253

**Published:** 2022-08-30

**Authors:** Yahya Mahamat‐Saleh, Marie Al‐Rahmoun, Gianluca Severi, Reza Ghiasvand, Marit B. Veierod, Saverio Caini, Domenico Palli, Edoardo Botteri, Carlotta Sacerdote, Fulvio Ricceri, Marko Lukic, Maria J. Sánchez, Valeria Pala, Rosario Tumino, Paolo Chiodini, Pilar Amiano, Sandra Colorado‐Yohar, María‐Dolores Chirlaque, Eva Ardanaz, Catalina Bonet, Verena Katzke, Rudolf Kaaks, Matthias B. Schulze, Kim Overvad, Christina C. Dahm, Christian S. Antoniussen, Anne Tjønneland, Cecilie Kyrø, Bas Bueno‐de‐Mesquita, Jonas Manjer, Malin Jansson, Anders Esberg, Nagisa Mori, Pietro Ferrari, Elisabete Weiderpass, Marie‐Christine Boutron‐Ruault, Marina Kvaskoff

**Affiliations:** ^1^ Paris‐Saclay University, UVSQ Inserm, Gustave Roussy, “Exposome and Heredity” team, CESP Villejuif France; ^2^ Oslo Centre for Biostatistics and Epidemiology Oslo University Hospital Oslo Norway; ^3^ Department of Research Cancer Registry of Norway, Oslo University Hospital Oslo Norway; ^4^ Oslo Centre for Biostatistics and Epidemiology, Department of Biostatistics Institute of Basic Medical Sciences, University of Oslo Oslo Norway; ^5^ Cancer Risk Factors and Life‐Style Epidemiology Unit, Institute for Cancer Research Prevention and Clinical Network (ISPRO) Florence Italy; ^6^ Section for Colorectal Cancer Screening Cancer Registry of Norway, Oslo University Hospital Oslo Norway; ^7^ Piedmont Reference Centre for Epidemiology and Cancer Prevention (CPO Piemonte) Turin Italy; ^8^ Epidemiology Unit ASL TO3 Piedmont Region Grugliasco Italy; ^9^ Faculty of Health Sciences, Department of Community Medicine University of Tromsø, The Arctic University of Norway Norway; ^10^ Escuela Andaluza de Salud Pública (EASP) Granada Spain; ^11^ Instituto de Investigación Biosanitaria ibs.GRANADA Granada Spain; ^12^ Centro de Investigación Biomédica en Red de Epidemiología y Salud Pública (CIBERESP) Madrid Spain; ^13^ Department of Preventive Medicine and Public Health University of Granada Granada Spain; ^14^ Epidemiology and Prevention Unit Fondazione IRCCS Istituto Nazionale dei Tumori di Milano Milan Italy; ^15^ Hyblean Association for Epidemiological Research AIRE—ONLUS Ragusa Italy; ^16^ Dipartimento di Salute Mentale e Fisica e Medicina Preventiva Università degli Studi della Campania “Luigi Vanvitelli” Naples Italy; ^17^ Ministry of Health of the Basque Government Sub‐Directorate for Public Health and Addictions of Gipuzkoa San Sebastian Spain; ^18^ Biodonostia Health Research Institute Epidemiology of Chronic and Communicable Diseases Group San Sebastián Spain; ^19^ Department of Epidemiology Murcia Regional Health Council, IMIB‐Arrixaca, Murcia University Murcia Spain; ^20^ Research Group on Demography and Health, National Faculty of Public Health University of Antioquia Medellín Colombia; ^21^ Navarra Public Health Institute Pamplona Spain; ^22^ IdiSNA, Navarra Institute for Health Research Pamplona Spain; ^23^ Unit of Nutrition and Cancer, Catalan Institute of Oncology—ICO Nutrition and Cancer Group, Bellvitge Biomedical Research Institute—(IDIBELL), L'Hospitalet de Llobregat Barcelona Spain; ^24^ Division of Cancer Epidemiology German Cancer Research Center (DKFZ) Heidelberg Germany; ^25^ Department of Molecular Epidemiology German Institute of Human Nutrition Potsdam‐Rehbruecke Nuthetal Germany; ^26^ Institute of Nutritional Science University of Potsdam Nuthetal Germany; ^27^ Department of Public Health Aarhus University Aarhus C Denmark; ^28^ Danish Cancer Society Research Center; Diet Genes and Environment Nutrition and Biomarkers (NAB) Copenhagen Denmark; ^29^ Centre for Nutrition Prevention and Health Services, National Institute for Public Health and the Environment (RIVM) Bilthoven The Netherlands; ^30^ Department of Surgery Skåne University Hospital Malmö Lund University Malmö Malmö Sweden; ^31^ Department of Surgery and Perioperative Sciences/Surgery Umeå University Umeå Sweden; ^32^ Department of Odontology Umeå University Umeå Sweden; ^33^ International Agency for Research on Cancer World Health Organization Lyon France

**Keywords:** alcohol, cohort studies, cutaneous melanoma, epidemiology, keratinocyte cancers

## Abstract

Experimental evidence suggests that alcohol induces cutaneous carcinogenesis, yet epidemiological studies on the link between alcohol intake and skin cancer have been inconsistent. The European Prospective Investigation into Cancer and Nutrition (EPIC) is a prospective cohort initiated in 1992 in 10 European countries. Alcohol intake at baseline and average lifetime alcohol intake were assessed using validated country‐specific dietary and lifestyle questionnaires. Hazard ratios (HRs) and 95% confidence intervals (CIs) were estimated in Cox models. A total of 14 037 skin cancer cases (melanoma: n = 2457; basal‐cell carcinoma (BCC): n = 8711; squamous‐cell carcinoma (SCC): n = 1928; unknown: n = 941) were identified among 450 112 participants (average follow‐up: 15 years). Baseline alcohol intake was positively associated with SCC (>15 vs 0.1‐4.9 g/day: HR = 1.44, 95% CI = 1.17‐1.77; *P*_trend_ = .001), BCC (HR = 1.12, 95% CI = 1.01‐1.23; *P*_trend_ = .04), and melanoma risks in men (HR = 1.17, 95% CI = 0.95‐1.44; *P*_trend_ = .17), while associations were more modest in women (SCC: HR = 1.09, 95% CI = 0.90‐1.30; *P*_trend_ = .13; BCC: HR = 1.08, 95% CI = 1.00‐1.17, *P*_trend_ = .03; melanoma: HR = 0.93, 95% CI = 0.80‐1.08, *P*_trend_ = .13). Associations were similar for lifetime alcohol intake, with an attenuated linear trend. Lifetime liquor/spirit intake was positively associated with melanoma (fourth vs first quartile: HR = 1.47, 95% CI = 1.08‐1.99; *P*_trend_ = .0009) and BCC risks in men (HR = 1.17, 95% CI = 1.04‐1.31; *P*_trend_ = .14). Baseline and lifetime intakes of wine were associated with BCC risk (HR = 1.25 in men; HR = 1.11‐1.12; in women). No statistically significant associations were found between beverage types and SCC risk. Intake of beer was not associated with skin cancer risk. Our study suggests positive relationships between alcohol intake and skin cancer risk, which may have important implications for the primary prevention of skin cancer.

## Abstract 2

What's new?

Drinking alcohol can make the skin more sensitive to sunlight and vulnerable to skin cancer. Here, the authors conducted a large prospective cohort study to evaluate whether alcohol consumption correlates with skin cancer risk. Among the 450 112 participants, there were 2457 cases of melanoma, 8711 of basal cell carcinoma, and 1928 of squamous cell carcinoma. There was a positive association between alcohol and all three cancer types, stronger in men than in women. The association varied somewhat by beverage type.

**Abbreviations**
BCC: basal‐cell carcinoma
CI: confidence interval
CUP: continuous update project
E3N: Etude Epidemiologique auprès de femmes de l'Education Nationale
EPIC: European Prospective Investigation into Cancer and Nutrition
HPFS: Health Professionals Follow‐Up Study
HR: hazard ratio
IARC: International Agency for Research on Cancer
KC: keratinocyte cancer
NHS: Nurses' Health Study
SCC: squamous‐cell carcinoma
UV: ultraviolet radiation
WCRF/AICR: World Cancer Research Fund/American Institute for Cancer Research
WHI: Women's Health Initiative

## 1 INTRODUCTION

Alcohol consumption is one of the major risk factors for the development of cancer and death from various cancer sites, causing approximately 740 000 new cancer cases in 2020 and 376 000 annual cancer deaths, thus representing 4.1% of all new cases of cancer^1^ and 4.9% of all cancer deaths worldwide.^2^ Alcohol may lead to the development of cancer through the metabolism of ethanol into acetaldehyde, which can in turn induce the production of oxidative stress by biding to specific proteins, thus increasing the production of reactive oxygen species.^3^ Alcohol consumption may also be related to the development of skin cancer—the most common type of cancer in white‐skinned populations,^4^, ^5^, ^6^ the incidence of which is rising.^5^, ^7^ While experimental evidence suggests that alcohol intake may increase skin photosensitivity to the sun,^8^ compromise the antioxidant defense system of the skin,^9^ and induce premature skin aging and cutaneous carcinogenesis,^8^ epidemiological evidence of the impact of alcohol intake on skin cancer remains inconclusive.

Several studies have examined the relationship between alcohol intake and skin cancer risk.^10^, ^11^, ^12^, ^13^, ^14^, ^15^, ^16^, ^17^, ^18^, ^19^ Three recent reviews are available on melanoma. A first review based on 14 case‐control and two cohort studies suggested a positive association between alcohol intake and melanoma risk in 2014.^20^ In 2015, a pooled analysis of eight case‐control studies in women showed a weak positive association that was similar across types of alcoholic beverages (10%‐14% increase in risk).^11^ In 2018, a meta‐analysis of 12 case‐control and six cohort studies showed a positive association between alcohol intake and melanoma risk, with a 29% increased risk in the highest vs the lowest alcohol intake.^21^ With regards to keratinocyte cancers, a 2017 meta‐analysis reported a positive but modest association between alcohol intake and risk of keratinocyte cancers (KCs) in a dose‐dependent manner for both basal‐cell carcinoma (BCC) and squamous‐cell carcinoma (SCC) (7% and 10% increased risks, respectively).^22^

Despite the evidence, the 2018 Continuous Update Project (CUP) of the World Cancer Research Fund/American Institute for Cancer Research (WCRF/AICR) report highlighted limited yet suggestive evidence that alcohol intake increases the risks of melanoma and BCC^23^; the panel suggested that further prospective research was required before providing public health recommendations on alcohol intake and skin cancer risk. Most previous studies were retrospective, and few were able to explore the type of beverage, which might be important to account for differences in alcohol concentration and drinking habits associated with each beverage. In addition, a majority of previous studies focused on baseline alcohol intake, and few studies collected information on alcohol intake across adulthood.

In our study, we investigated the relationships between alcohol consumption (at baseline and over lifetime) and skin cancer risk, taking into account the types of alcoholic beverage, within the European Prospective Investigation into Cancer and Nutrition (EPIC) cohort study.

## 2 METHODS

### 2.1 EPIC participants

EPIC is an ongoing multicenter prospective cohort designed to investigate the relations between dietary habits, nutritional status and various lifestyle/environmental factors, and the incidence of cancer and other chronic diseases.^24^ Briefly, the cohort recruited 521 448 participants aged 25‐70 years at inclusion and recruited in 23 centers from 10 European countries between 1992 and 2000: Denmark, France, Italy, Spain, The Netherlands, Greece, Germany, Sweden, Norway, and the United Kingdom. Participants eligible for the subcohorts were generally selected from the general population of a specific geographical area, except for the French cohort that included members of a national health scheme primarily covering teachers; the Utrecht and Florence cohorts that were based on women who underwent breast cancer screening; cohorts from the Turin, Ragusa, and Spain centers, which recruited mostly blood donors; and the Oxford center that recruited a high proportion of health‐conscious individuals. The rationale, complete methods, and study design have been described in detail elsewhere.^24^

### 2.2 Exposure and covariate assessment

Dietary intakes, including alcohol intake over the 12 months before recruitment, were assessed using validated country‐specific dietary and lifestyle questionnaires designed to reflect local dietary patterns at baseline. In each country, participants reported the number of glasses of beer, cider, wine, sweet liquor, distilled spirits, or fortified wines consumed per day during the 12 months prior to recruitment and over. Country‐specific intake was calculated based on estimated average glass volume and ethanol content for each type of alcoholic beverage, using information collected through 24‐hour dietary recalls from a subgroup of the cohort.

Baseline alcohol intake was calculated as the sum of the number of glasses of each type of alcoholic beverage (beer and/or cider, wine, sweet liquors and/or distilled spirits, and wines) consumed per day. Due to the regional specificity of alcohol drinking, alcohol content and glass volumes were heterogeneous across EPIC countries, therefore alcohol intake was calculated from country‐specific questionnaires, which have previously been standardized across countries. Average lifetime alcohol intake was assessed through the number of glasses consumed per week at 20, 30, 40, and 50 years of age and at baseline. This information was collected in most centers, except for Naples (Italy), Bilthoven (The Netherlands), Sweden, and Norway. Information on smoking status, physical activity during leisure time, educational level, and anthropometric factors was obtained using lifestyle questionnaires in all centers.

### 2.3 Follow‐up and identification of cancer cases

Incident skin cancer cases were identified through a combination of several methods, including record linkage with population‐based cancer registries, health insurance records, pathology registries, and active follow‐up of study subjects. During follow‐up, mortality data or vital status was obtained from cancer or mortality registries at the regional or national level. Skin cancer events were mostly ascertained through population‐based cancer registries or pathology reports (96% of cases; melanoma: 92%; BCC: 96%; SCC: 99%), and a small proportion (4%) was identified from hospital admission and discharge records or national/regional mortality registries. Although melanomas are more accurately recorded in cancer registries, registration of KCs, especially BCCs, was often incomplete in some centers because these cancers are not systematically recorded in cancer registries. Follow‐up began on the date of recruitment and ended on the date of skin cancer diagnosis, death, emigration/loss of follow‐up, or completion of the last returned questionnaire, whichever came first. Cancer incidence data were coded according to the International Classification of Diseases for Oncology (ICD‐O‐3). Cancer cases were defined as subjects with a first primary incident skin cancer (including KC; C44). Information on stage, site, morphology, and grade of melanoma was collected from each center, where possible. In this current study, skin cancer cases combine melanoma, BCC, SCC, and unknown skin cancer type.

### 2.4 Study sample

From the cohort of 521 448 women and men, we first excluded subjects with prevalent cancer cases (including KCs) or those with missing data on date of diagnosis and follow‐up information (n = 29 456), and those with missing information on lifestyle factors (n = 6259) or extreme energy intake values (<first and > 99th percentiles of the distribution) (n = 9573). We further excluded subjects from the Greece cohort (n = 26 048) due to data restriction issues, leaving a final sample of 450 112 participants for analysis of baseline alcohol intake. Analysis of lifetime alcohol intake was based on 363 310 EPIC participants after exclusion of 112 850 participants—from Norway, Sweden, Naples, and Bilthoven—for whom data on lifetime alcohol was missing.

### 2.5 Statistical analysis

Hazard ratios (HRs) and 95% confidence intervals (CIs) for the association between alcohol intake and risks of overall skin cancer, melanoma, BCC, or SCC were estimated using Cox proportional hazards regression with age as the time scale. The analyses were conducted separately in men and women to consider gender differences in alcohol drinking behaviors and alcohol metabolism. We first investigated associations between baseline and lifetime alcohol intake and skin cancer risk. Secondly, we assessed associations with different types of alcoholic beverages. Baseline and average lifetime intakes were modeled as categorical variables (nondrinker, >0‐4.9, 5‐15, and >15 g/day), using the “moderate consumption” group (>0‐4.9 g/day) as the reference category to ensure a sufficiently large number of cases. Analyses by type of alcoholic beverage were performed among alcohol consumers. For each type of beverage, consumers of that beverage were categorized into quartiles. Tests for linear trends were performed using an ordinal variable across alcohol intake categories. Cox regression models were first stratified by study center to control for different follow‐up procedures and questionnaire design across centers, and by age at recruitment (in 1‐year intervals), then additionally adjusted for body mass index, smoking status, education, physical activity level, and total energy intake. For analyses on type of alcoholic beverage, models were additionally mutually adjusted for intake of other alcoholic beverages (quartiles, Q_x_). In addition, we performed analyses considering alcohol intake at different ages.

Sensitivity analyses were performed by additionally adjusting for hours of recreational physical activity in summer (number of hours of walking, cycling, gardening, and physical exercise in a typical week during the past year), which we used as a proxy for recreational sun exposure. Also, we evaluated potential effect modification by recreational physical activity in summer using interaction terms, and comparing the associated Wald test statistics to a chi‐square distribution with degrees of freedom equal to the number of categories minus one, not including the nondrinker category. In addition, we conducted separate analyses by country, using alcohol as a continuous variable (12 g/day increments in intake). We further assessed associations by tumor anatomical site (head, neck and extremities, and trunk)^25^, ^26^ and, for melanoma, histologic subtype (superficial spreading, lentigo maligna, nodular, acro‐lentiginous, and other), using competing‐risk analysis, excluding cases with missing information on tumor characteristics for these analyses. Homogeneity tests were performed using Wald chi‐square tests to compare estimates by tumor sites and types. All statistical analyses were performed using the SAS software (version 9.4, SAS Institute), and all significance tests were two‐sided, with *P*‐values less than 5% considered statistically significant.

## 3 RESULTS

A total of 14 037 skin cancer cases (melanoma: n = 2457; BCC: n = 8711; SCC: n = 1928, unknown type: n = 941) were identified among 450 112 participants within an average of 15 years of follow‐up. Baseline intake of alcohol was the highest in Denmark and Spain, followed by Italy and Germany, and the lowest in Sweden and Norway. Median alcohol intake at baseline was 7.2 g/day, mainly from wine (4.6 g/day) (Table 1). Subjects with an intake of alcohol higher than 15 g/day were younger and more likely to be current smokers, to have higher education and physical activity levels, and a higher intake of total energy compared to nondrinkers (Table 2). However, they were less likely to be obese. Men were more likely to drink alcohol than women.

**TABLE 1:** Cohort characteristics by country, European Prospective Investigation into Cancer and Nutrition (EPIC) cohort study (n = 450 112)

							Baseline total alcohol intake (g/d)^a^		Average lifetime alcohol intake (g/d)		Baseline wine intake (g/d)	Baseline beer intake (g/d)	Baseline spirits/liquors intake (g/d)
	Cohort size	Skin cancer cases	Recruitment period range	Age at recruitment (years)	Women (%)	Length of follow‐up (years)	Nondrinkers (%)	Median (P10‐P90)^a^	Nondrinkers (%)	Median (P10‐P90)^a^	Median (P10‐P90)^a^	Median (P10‐P90)^a^	Median (P10‐P90)^a^
France	67 403	1800	1993‐1997	51.0	100.0	14.7	13.6	7.8 (0.9‐30.46)	10.4	4.5 (0.2‐17.9)	6.8 (0.9‐28.4)	0.9 (0.1‐6.1)	1.1 (0.2‐5.3)
Italy	44 545	890	1992‐1998	51.0	68.5	14.9	16.5	11.8 (0.4‐41.5)	8.7	7.8 (0.7‐36.1)	11.8 (0.2‐35.5)	0.2 (0.2‐3.4)	0.2 (0.2‐5.2)
Spain	39 989	818	1992‐1996	49.0	62.1	16.7	37.6	12.5 (0.9‐54.4)	23.2	11.4 (0.7‐67.3)	12.8 (0.8‐50.6)	2.7 (0.6‐13.5)	3.5 (0.9‐15.6)
The UK	75 416	3362	1993‐2001	49.0	69.7	15.9	6.1	5.8 (0.4‐24.9)	1.6	6.9 (0.3‐25.3)	1.7 (0.1‐11.7)	0.7 (0.1‐7.5)	0.5 (0.1‐3.3)
The Netherlands	36 539	544	1994‐1997	51.0	73.7	15.1	14.4	7.3 (0.5‐32.6)	10.1	5.4 (1.3‐18.4)	2.9 (0.2‐17.2)	2.4 (0.1‐23.5)	0.5 (0.02‐9.1)
Germany	48 557	769	1994‐1998	51.0	56.4	11.5	4.1	9.4 (1.0‐42.5)	1.0	8.5 (1.4‐37.9)	3.6 (0.4‐18.3)	3.0 (0.2‐28.3)	0.5 (0.1‐3.0)
Denmark	55 014	4726	1993‐1997	56.0	52.2	16.2	2.2	13.4 (1.9‐48.2)	0.9	10.6 (2.2‐32.2)	5.1 (1‐29.8)	5.3 (0.4‐30.8)	0.9 (0.2‐4.6)
Norway	33 975	281	1998	48.0	100.0	14.1	20.7	2.2 (0.5‐8.8)	_	_	0.9 (0.4‐4.8)	1.2 (0.5‐2.1)	_
Sweden	48 674	847	1991‐1996	51.0	54.2	18.0	11.8	4.4 (0.4‐19.1)	_	_	1.8 (0.1‐9.4)	1.7 (0.1‐6.8)	1.0 (0.1‐6.7)
All	450 112	14 037	1991‐2001	51.0	70.8	14.9	12.8	7.2 (0.7‐35.2)	6.9	7.5 (0.6‐31.2)	4.6 (0.2‐24.3)	1.4 (0.1‐12.7)	0.5 (0.1‐5.3)

^a^ Median (10th‐90th percentiles) calculated in drinkers only.

**TABLE 2:** Baseline characteristics of study participants according to baseline total alcohol consumption, EPIC cohort (n = 450 112)

	Baseline total alcohol intake, g/d			
	Nondrinkers	>0.0‐4.9	5.0‐15.0	>15.0
*Men*				
Participants (n, %)	8100 (6.2)	28 858 (22.0)	34 860 (26.5)	59 608 (45.3)
Age at recruitment (years; mean, SD)^a^	53.7 (10.3)	50.1 (11.4)	51.9 (10.5)	52.6 (8.6)
Smoking (%)				
Never smoker	54.8	44.0	38.1	25.9
Former smoker	25.8	32.0	36.7	39.4
Current smoker	30.0	22.4	24.0	34.0
Unknown	1.4	1.6	1.2	0.7
Educational level (%)				
None/primary	48.4	31.1	28.2	32.3
Technical/secondary school	33.7	41.2	39.2	36.5
University degree	15.1	22.5	29.2	29.6
Unknown	2.8	5.2	3.4	1.6
Physical activity level (%)				
Inactive	16.9	17.3	23.8	28.5
Moderately inactive	36.5	25.2	29.0	30.6
Moderately active	29.3	23.6	25.9	29.4
Active	7.4	6.8	7.5	8.8
Missing	9.9	27.1	13.8	2.7
Body mass index, kg/m^2^				
<18.5	0.6	0.6	0.4	0.3
18.5‐24.9	32.8	41.3	39.8	33.1
25‐29.9	48.6	45.1	47.4	50.7
≥30	18.0	13.0	12.4	15.9
Total energy intake (kcal/day)^a^	2327.3 (683.5)	2245.1 (664.0)	2331.0 (626.7)	2562.35 (646.8)
Baseline total alcohol intake (g/d)^b^	0 (0‐0)	1.9 (0.4‐4.3)	9.3 (5.8‐13.5)	32.2 (17.3‐67.2)
Average lifetime alcohol intake (g/d)^b^	5.4 (0.0‐62.6)	4.8 (0.4‐22.4)	11.1 (4.6‐28.3)	28.0 (12.4‐65.7)
Baseline wine intake (g/d)^b^	0 (0‐0)	0.2 (0‐1.8)	3.6 (0‐9.4)	11.6 (0.5‐47.4)
Lifetime wine intake (g/d)^b^	0.2 (0.0‐21.9)	0.9 (0.01‐7.2)	3.2 (0.3‐12.6)	9.5 (1.00‐42.9)
Baseline beer intake (g/d)^b^	0 (0‐0)	0.6 (0‐2.7)	3.7 (0.1‐8.2)	7.9 (0‐39.7)
Lifetime beer intake (g/d)^b^	0.8 (0.0‐22.4)	1.4 (0.02‐8.5)	3.8 (0.4‐12.6)	7.5 (0.4‐28.4)
Baseline liquor and spirits intakes (g/d)^b^	0 (0‐0)	0.1 (0‐1.6)	0.5 (0‐3.7)	1.5 (0‐11.0)
Lifetime liquor and spirits intakes (g/d)^b^	0 (0‐15.5)	0.5 (0‐5.8)	1.4 (0‐7.8)	2.8 (0.02‐13.8)
*Women*				
Participants (n, %)	49 341 (15.5)	128 310 (40.3)	87 077 (27.3)	53 958 (16.9)
Age at recruitment (years; mean, SD)^a^	51.8 (8.9)	50.1 (10.0)	50.5 (9.9)	51.6 (8.6)
Smoking (%)				
Never smoker	63.8	56.8	53.5	44.7
Former smoker	15.6	22.6	26.2	27.8
Current smoker	18.3	18.4	18.1	25.3
Unknown	2.3	2.2	2.2	2.2
Educational level (%)				
None/primary	48.6	25.5	20.3	19.1
Technical/secondary school	36.3	48.9	47.7	47.1
University degree	13.1	20.3	27.7	31.0
Unknown	2.0	5.3	4.3	2.8
Physical activity level (%)				
Inactive	6.7	9.8	15.4	17.8
Moderately inactive	24.6	26.2	32.8	37.3
Moderately active	43.5	31.8	34.2	35.1
Active	7.0	7.4	8.2	8.0
Missing	18.2	24.8	9.4	1.8
Body mass index, kg/m^2^ (%)				
<18.5	2.0	2.2	2.0	1.8
18.5‐24.9	44.8	52.7	62.7	63.7
25‐29.9	33.2	33.2	26.9	26.9
≥30	20.0	11.9	8.4	7.6
Total energy intake (kcal/day)^a^	1846.3 (537.1)	1856.2 (522.6)	1972.9 (521.4)	2148.9 (554.1)
Baseline total alcohol intake (g/d)^b^	0 (0‐0)	1.5 (0.3‐3.9)	8.9 (5.6‐13.1)	24.4 (16.5‐46.9)
Average lifetime alcohol intake (g/d)^b^	0 (0‐3.8)	1.6 (0.1‐7.1)	6.5 (2.4‐15.0)	14.7 (7.1‐29.4)
Baseline wine intake (g/d)^b^	0 (0‐0)	0.8 (0‐2.6)	5.2 (1.5‐11.7)	19.4 (5.1‐36.0)
Lifetime wine intake (g/d)^b^	0 (0‐1.7)	0.6 (0‐3.7)	3.9 (0.9‐9.5)	9.9 (3.1‐21.5)
Baseline beer intake (g/d)^b^	0 (0‐0)	0.1 (0‐1.4)	0.5 (0‐5.1)	0.7 (0‐11.5)
Lifetime beer intake (g/d)^b^	0 (0‐0.6)	0.03 (0‐1.3)	0.3 (0‐3.6)	0.6 (0‐6.7)
Baseline liquor and spirits intakes (g/d)^b^	0 (0‐0)	0 (0‐0.5)	0.07 (0‐2.4)	0.3 (0‐5.7)
Lifetime liquor and spirits intakes (g/d)^b^	0 (0‐0.1)	0.01 (0‐1.6)	0.1 (0‐3.4)	0.4 (0‐5.3)

Abbreviations: EPIC: European Prospective Investigation into Cancer and Nutrition.

^a^ Mean (SD).

^b^ Median (10th‐90th percentiles).

### 3.1 Baseline and average lifetime alcohol intake

In men, baseline alcohol intake was positively and linearly associated with skin cancer risk (HR = 1.16, 95% CI = 1.07‐1.26 for >15 g/day vs 0.1‐4.9 g/day, *P*_trend_ = .0002), particularly with SCC (HR = 1.44, 95% CI = 1.17‐1.77, *P*_trend_ = .001) and BCC (HR = 1.12, 95% CI = 1.01‐1.23, *P*_trend_ = .04) risks (Table 3). We found a positive but not statistically significant association with melanoma risk (HR = 1.17, 95% CI = 0.95‐1.44, *P*_trend_ = .17), although with no heterogeneity across skin cancer types (*P*_heterogeneity_ = .25). Similarly, in men, we found a positive and linear association between average lifetime alcohol intake and skin cancer risk (HR = 1.15, 95% CI = 1.05‐1.26 for >15 g/day vs 0.1‐4.9 g/day, *P*_trend_ = .01), particularly with SCC (HR = 1.29, 95% CI = 1.00‐1.67, *P*_trend_ = .02) and BCC (HR = 1.14, 95% CI = 1.02‐1.27, *P*_trend_ = .06) (melanoma: HR = 1.10, 95% CI = 0.84‐1.44, *P*_trend_ = .67; *P*_heterogeneity_ = .43).

**TABLE 3:** Hazard ratios (HRs) and 95% confidence intervals (CIs) for baseline and lifetime total alcohol intake and risk of skin cancer, by sex, EPIC cohort (n = 450 112)

	Skin cancer			Melanoma			BCC			SCC		
		Age‐adjusted^a^	Multivariable‐adjusted^b^		Age‐adjusted^a^	Multivariable‐adjusted^b^		Age‐adjusted^a^	Multivariable‐adjusted^b^		Age‐adjusted^a^	Multivariable‐adjusted^b^
	Cases	HR (95% CI)	HR (95% CI)	Cases	HR (95% CI)	HR (95% CI)	Cases	HR (95% CI)	HR (95% CI)	Cases	HR (95% CI)	HR (95% CI)
*Men*												
Baseline total alcohol intake (g/d)												
Nondrinkers	271	0.99 (0.86‐1.13)	1.00 (0.87‐1.15)	46	1.03 (0.74‐1.45)	1.06 (0.76‐1.49)	163	0.99 (0.83‐1.19)	1.00 (0.84‐1.20)	56	1.08 (0.79‐1.48)	1.09 (0.79‐1.49)
>0‐4.9	934	1.00 (reference)	1.00 (reference)	158	1.00 (reference)	1.00 (reference)	574	1.00 (reference)	1.00 (reference)	143	1.00 (reference)	1.00 (reference)
5‐14.9	1541	1.15 (1.06‐1.25)	1.14 (1.05‐1.24)	230	1.11 (0.90‐1.36)	1.10 (0.89‐1.35)	1006	1.14 (1.03‐1.27)	1.13 (1.02‐1.25)	254	1.37 (1.11‐1.69)	1.36 (1.10‐1.68)
>15	2721	1.17 (1.08‐1.27)	1.16 (1.07‐1.26)	382	1.16 (0.95‐1.42)	1.17 (0.95‐1.44)	1870	1.13 (1.02‐1.25)	1.12 (1.01‐1.23)	365	1.44 (1.17‐1.77)	1.44 (1.17‐1.77)
*P*_trend_		<.0001	.0002		.16	.17		.02	.04		.0008	.001
Average lifetime alcohol intake (g/d)^c^												
Nondrinkers	52	1.00 (0.75‐1.33)	1.00 (0.75‐1.33)	8	1.54 (0.74‐3.23)	1.54 (0.74‐3.24)	37	0.98 (0.70‐1.38)	0.98 (0.70‐1.38)	5	0.65 (0.26‐1.62)	0.65 (0.26‐1.61)
>0‐4.9	683	1.00 (reference)	1.00 (reference)	76	1.00 (reference)	1.00 (reference)	480	1.00 (reference)	1.00 (reference)	101	1.00 (reference)	1.00 (reference)
5‐14.9	1663	1.13 (1.03‐1.24)	1.13 (1.03‐1.24)	174	1.02 (0.78‐1.35)	1.03 (0.78‐1.36)	1247	1.16 (1.04‐1.29)	1.16 (1.04‐1.29)	193	1.13 (0.88‐1.45)	1.14 (0.88‐1.46)
>15	2425	1.12 (1.02‐1.22)	1.15 (1.05‐1.26)	300	1.06 (0.81‐1.39)	1.10 (0.84‐1.44)	1773	1.11 (1.00‐1.23)	1.14 (1.02‐1.27)	268	1.24 (0.97‐1.60)	1.29 (1.00‐1.67)
*P*_trend_		.06	.01		.91	.67		.21	.06		.04	.02
*Women*												
Baseline total alcohol intake (g/d)												
Nondrinkers	1042	1.01 (0.93‐1.09)	1.02 (0.95‐1.11)	246	1.06 (0.91‐1.24)	1.09 (0.93‐1.27)	537	1.01 (0.91‐1.12)	1.02 (0.92‐1.14)	173	0.95 (0.79‐1.15)	0.98 (0.81‐1.18)
>0‐4.9	2971	1.00 (reference)	1.00 (reference)	659	1.00 (reference)	1.00 (reference)	1656	1.00 (reference)	1.00 (reference)	401	1.00 (reference)	1.00 (reference)
5‐14.9	2767	1.11 (1.06‐1.17)	1.08 (1.02‐1.14)	466	1.00 (0.89‐1.13)	0.98 (0.87‐1.11)	1741	1.15 (1.07‐1.23)	1.10 (1.03‐1.18)	353	1.18 (1.02‐1.37)	1.14 (0.99‐1.32)
>15	1790	1.10 (1.04‐1.17)	1.05 (0.99‐1.12)	270	0.96 (0.83‐1.11)	0.93 (0.80‐1.08)	1164	1.14 (1.05‐1.23)	1.08 (1.00‐1.17)	183	1.14 (0.95‐1.36)	1.09 (0.90‐1.30)
*P*_trend_		.0003	.08		.35	.13		.0002	.03		.02	.13
Average lifetime alcohol intake (g/d)^c^												
Nondrinkers	546	1.03 (0.93‐1.13)	1.04 (0.95‐1.15)	102	1.18 (0.95‐1.48)	1.20 (0.95‐1.50)	323	1.02 (0.90‐1.16)	1.04 (0.92‐1.18)	62	0.88 (0.66‐1.17)	0.91 (0.69‐1.21)
>0‐4.9	3348	1.00 (reference)	1.00 (reference)	514	1.00 (reference)	1.00 (reference)	2136	1.00 (reference)	1.00 (reference)	385	1.00 (reference)	1.00 (reference)
5‐14.9	2654	1.07 (1.02‐1.13)	1.04 (0.99‐1.10)	375	1.03 (0.90‐1.18)	1.02 (0.89‐1.16)	1801	1.09 (1.02‐1.16)	1.06 (0.99‐1.13)	265	1.08 (0.92‐1.27)	1.04 (0.88‐1.22)
>15	1059	1.10 (1.03‐1.18)	1.06 (0.99‐1.14)	171	1.13 (0.95‐1.35)	1.10 (0.92‐1.32)	724	1.14 (1.04‐1.24)	1.10 (1.01‐1.20)	82	0.98 (0.76‐1.25)	0.93 (0.72‐1.19)
*P*_trend_		.005	.15		.67	.94		.002	.05		.41	.91

Abbreviations: BCC, basal‐cell carcinoma; CI, confidence intervals; EPIC, European Prospective Investigation into Cancer and Nutrition; HR, hazard ratio; SCC, squamous‐cell carcinoma.

^a^ Age‐adjusted: adjusted for age and stratified by age at recruitment and study center.

^b^ Multivariable‐adjusted model: stratified by age at recruitment and study center, and adjusted for educational level (none, primary school, technical/professional school, secondary school, university or higher degree), body mass Index (BMI; <25, 25‐29, or ≥30 kg/m^2^), smoking (never, former, and current), physical activity level (metabolic equivalent of task [MET] hour/week), and total energy intake (continuous).

^c^ Analysis of lifetime alcohol intake was based on 363 310 EPIC participants after exclusion of participants from Norway, Sweden, Naples, and Bilthoven—for whom data on lifetime alcohol was missing.

In women, there was a small and nonsignificant association between baseline intake of alcohol and skin cancer risk (HR = 1.05, 95% CI = 0.99‐1.12, *P*_trend_ = .08) (Table 3). The association was statistically significant for BCC (HR = 1.08, 95% CI = 1.00‐1.17, *P*_trend_ = .03), although there was no heterogeneity across skin cancer types (*P*_heterogeneity_ = .21). Similar results were observed for lifetime average alcohol intake.

These results were unchanged after further adjustment for hours of recreational sun exposure during outdoor physical activity in summer (data not shown).

### 3.2 Types of alcoholic beverage

In men, while no association was observed between baseline intake of spirits/liquors and skin cancer risk (HR = 1.07, 95% CI = 0.93‐1.22 for high intake (Q4) vs low (Q1), *P*_trend_ = .09), lifetime intake of spirits/liquors was positively associated with a higher risk of skin cancer (HR = 1.18, 95% CI = 1.07‐1.30, *P*_trend_ = .003), particularly with melanoma (HR = 1.47, 95% CI = 1.08‐1.99, *P*_trend_ = .0009) and BCC (HR = 1.17, 95% CI = 1.04‐1.31, *P*_trend_ = .14; *P*_heterogeneity_ = .15) (Table 4). Baseline intake of wine was linearly and positively associated with skin cancer risk (HR = 1.22, 95% CI = 1.10‐1.35, *P*_trend_ = .0001), in particular with BCC (HR = 1.25, 95% CI = 1.10‐1.42, *P*_trend_ = .004). The results were generally stable when average lifetime intake of wine was investigated. We found a positive association between baseline beer intake and skin cancer risk in the second and third quartiles of intake, particularly with BCC risk (Q2: HR = 1.21, 95% CI = 1.04‐1.41 and Q3: HR = 1.20, 95% CI = 1.04‐1.40), although there was no significant association in the fourth quartile and no linear trend. Similar results were obtained with average lifetime intake of beer with a positive association in the third quartile only.

**TABLE 4:** Hazard ratios (HRs) and 95% confidence intervals (CIs) for intake of baseline and lifetime total alcohol and risk of skin cancer, according to different types of alcoholic beverage and sex, EPIC cohort (n = 450 112)^a^

	Men				Women			
	Skin cancer	Melanoma	BCC	SCC	Skin cancer	Melanoma	BCC	SCC
	HR^a^ (95% CI)	HR^a^ (95% CI)	HR^a^ (95% CI)	HR^a^ (95% CI)	HR^a^ (95% CI)	HR^a^ (95% CI)	HR^a^ (95% CI)	HR^a^ (95% CI)
Baseline intake of liquor and spirits (g/d)^b^								
Nondrinkers	1.01 (0.87‐1.17)	1.11 (0.78‐1.56)	1.03 (0.85‐1.24)	1.06 (0.72‐1.57)	0.97 (0.88‐1.07)	0.92 (0.75‐1.14)	0.97 (0.85‐1.12)	0.96 (0.74‐1.24)
Q1 (>0‐0.18)	1.00 (reference)	1.00 (reference)	1.00 (reference)	1.00 (reference)	1.00 (reference)	1.00 (reference)	1.00 (reference)	1.00 (reference)
Q2 (>0.18‐0.55)	0.92 (0.79‐1.07)	1.03 (0.71‐1.50)	0.94 (0.77‐1.13)	0.87 (0.55‐1.37)	1.04 (0.94‐1.15)	1.05 (0.84‐1.30)	1.03 (0.91‐1.18)	1.06 (0.81‐1.38)
Q3 (>0.55‐1.84)	1.02 (0.89‐1.17)	1.09 (0.79‐1.50)	1.03 (0.87‐1.23)	1.06 (0.73‐1.55)	1.08 (0.97‐1.19)	1.05 (0.84‐1.32)	1.09 (0.95‐1.25)	1.13 (0.85‐1.49)
Q4 (>1.84)	1.07 (0.93‐1.22)	1.28 (0.92‐1.77)	1.07 (0.90‐1.28)	1.03 (0.71‐1.49)	1.08 (0.97‐1.19)	0.97 (0.77‐1.24)	1.10 (0.96‐1.26)	1.08 (0.82‐1.42)
*P*_trend_	.09	.13	.2	.93	.002	.3	.007	.14
Average lifetime intake of liquor and spirits (g/d)^b^ ^,^ ^c^								
Nondrinkers	1.10 (0.96‐1.26)	0.88 (0.57‐1.34)	1.20 (1.03‐1.41)	0.89 (0.60‐1.32)	0.97 (0.89‐1.05)	0.95 (0.78‐1.16)	0.98 (0.89‐1.08)	0.89 (0.70‐1.14)
Q1 (>0‐0.13)	1.00 (reference)	1.00 (reference)	1.00 (reference)	1.00 (reference)	1.00 (reference)	1.00 (reference)	1.00 (reference)	1.00 (reference)
Q2 (>0.13‐0.99)	1.09 (0.97‐1.22)	1.23 (0.87‐1.74)	1.12 (0.97‐1.28)	0.90 (0.63‐1.28)	1.00 (0.93‐1.08)	0.94 (0.77‐1.16)	1.02 (0.93‐1.12)	0.93 (0.73‐1.19)
Q3 (>0.99‐3.08)	1.18 (1.07‐1.30)	1.10 (0.80‐1.51)	1.21 (1.07‐1.36)	1.07 (0.79‐1.45)	1.03 (0.95‐1.11)	1.15 (0.94‐1.41)	1.03 (0.94‐1.13)	0.95 (0.75‐1.20)
Q4 (>3.08)	1.18 (1.07‐1.30)	1.47 (1.08‐1.99)	1.17 (1.04‐1.31)	0.95 (0.70‐1.28)	1.03 (0.94‐1.13)	1.15 (0.91‐1.46)	1.02 (0.91‐1.14)	1.04 (0.79‐1.38)
*P*_trend_	.003	.0009	.14	.70	.12	.05	.3	.47
Baseline intake of wine (g/d)^b^								
Nondrinkers	1.06 (0.94‐1.20)	0.93 (0.70‐1.24)	1.13 (0.96‐1.33)	0.92 (0.69‐1.21)	1.04 (0.96‐1.13)	1.12 (0.94‐1.32)	1.06 (0.94‐1.18)	0.91 (0.74‐1.12)
Q1 (>0‐0.98)	1.00 (reference)	1.00 (reference)	1.00 (reference)	1.00 (reference)	1.00 (reference)	1.00 (reference)	1.00 (reference)	1.00 (reference)
Q2 (>0.98‐4.64)	1.11 (1.00‐1.24)	0.94 (0.73‐1.22)	1.19 (1.04‐1.36)	0.98 (0.74‐1.28)	1.03 (0.96‐1.11)	0.94 (0.80‐1.11)	1.08 (0.98‐1.20)	0.98 (0.80‐1.20)
Q3 (>4.64‐10.03)	1.18 (1.08‐1.30)	0.98 (0.76‐1.27)	1.25 (1.11‐1.42)	1.08 (0.84‐1.40)	1.08 (1.00‐1.16)	1.03 (0.88‐1.22)	1.09 (0.99‐1.20)	1.13 (0.92‐1.37)
Q4 (>10.03)	1.22 (1.10‐1.35)	1.15 (0.89‐1.50)	1.25 (1.10‐1.42)	1.12 (0.86‐1.47)	1.08 (1.00‐1.16)	0.94 (0.78‐1.12)	1.12 (1.02‐1.24)	1.04 (0.83‐1.30)
*P*_trend_	.0001	.14	.004	.08	.09	.13	.07	.09
Average lifetime intake of wine (g/d)^b^ ^,^ ^c^								
Nondrinkers	1.13 (0.98‐1.32)	1.21 (0.78‐1.89)	1.11 (0.93‐1.33)	1.18 (0.80‐1.73)	1.09 (0.99‐1.19)	1.32 (1.06‐1.64)	1.05 (0.94‐1.19)	0.98 (0.76‐1.26)
Q1 (>0‐1.14)	1.00 (reference)	1.00 (reference)	1.00 (reference)	1.00 (reference)	1.00 (reference)	1.00 (reference)	1.00 (reference)	1.00 (reference)
Q2 (>1.14‐3.47)	1.18 (1.07‐1.30)	1.13 (0.84‐1.52)	1.18 (1.05‐1.32)	1.10 (0.83‐1.45)	1.06 (0.99‐1.13)	1.23 (1.03‐1.47)	1.03 (0.94‐1.12)	0.95 (0.78‐1.17)
Q3 (>3.47‐8.65)	1.22 (1.10‐1.35)	1.27 (0.94‐1.70)	1.21 (1.08‐1.36)	1.21 (0.91‐1.60)	1.09 (1.02‐1.17)	1.11 (0.93‐1.33)	1.07 (0.98‐1.16)	1.17 (0.94‐1.45)
Q4 (>8.65)	1.26 (1.14‐1.40)	1.29 (0.95‐1.75)	1.25 (1.11‐1.41)	1.33 (0.99‐1.78)	1.09 (1.01‐1.17)	1.13 (0.92‐1.38)	1.11 (1.01‐1.22)	0.86 (0.65‐1.12)
*P*_trend_	.0001	.17	.002	.12	.17	.70	.07	.99
Baseline intake of beer (g/d)^b^								
Nondrinkers	1.06 (0.92‐1.22)	1.13 (0.80‐1.61)	1.12 (0.94‐1.32)	0.97 (0.66‐1.43)	0.97 (0.90‐1.05)	1.05 (0.88‐1.26)	0.97 (0.87‐1.07)	0.94 (0.74‐1.19)
Q1 (>0‐0.41)	1.00 (reference)	1.00 (reference)	1.00 (reference)	1.00 (reference)	1.00 (reference)	1.00 (reference)	1.00 (reference)	1.00 (reference)
Q2 (>0.41‐1.37)	1.15 (1.01‐1.30)	0.91 (0.65‐1.28)	1.21 (1.04‐1.41)	0.97 (0.66‐1.43)	1.02 (0.94‐1.11)	1.15 (0.95‐1.39)	1.01 (0.91‐1.12)	0.99 (0.76‐1.27)
Q3 (>1.37‐5.28)	1.19 (1.06‐1.35)	1.26 (0.92‐1.72)	1.20 (1.04‐1.40)	1.13 (0.79‐1.62)	0.99 (0.91‐1.08)	1.09 (0.90‐1.33)	0.96 (0.86‐1.07)	0.98 (0.75‐1.29)
Q4 (>5.28)	1.09 (0.97‐1.24)	1.01 (0.73‐1.38)	1.11 (0.95‐1.29)	1.24 (0.87‐1.78)	1.00 (0.90‐1.10)	1.10 (0.86‐1.40)	1.02 (0.91‐1.15)	0.87 (0.62‐1.22)
*P*_trend_	.32	.78	.76	.05	.49	.40	.52	.96
Average lifetime intake of beer (g/d)^b^ ^,^ ^c^								
Nondrinkers	1.02 (0.84‐1.23)	1.34 (0.77‐2.30)	1.10 (0.88‐1.38)	0.66 (0.40‐1.11)	1.03 (0.95‐1.10)	1.12 (0.93‐1.35)	1.04 (0.95‐1.14)	0.97 (0.77‐1.23)
Q1 (>0‐0.33)	1.00 (reference)	1.00 (reference)	1.00 (reference)	1.00 (reference)	1.00 (reference)	1.00 (reference)	1.00 (reference)	1.00 (reference)
Q2 (>0.33‐1.35)	1.02 (0.87‐1.18)	1.20 (0.76‐1.89)	1.09 (0.91‐1.30)	0.78 (0.52‐1.16)	1.09 (1.02‐1.18)	1.21 (1.00‐1.47)	1.10 (1.01‐1.20)	0.95 (0.74‐1.21)
Q3 (>1.35‐4.89)	1.15 (1.00‐1.32)	1.15 (0.75‐1.79)	1.20 (1.01‐1.42)	1.03 (0.72‐1.48)	1.00 (0.93‐1.09)	1.12 (0.90‐1.38)	1.01 (0.92‐1.11)	0.90 (0.68‐1.19)
Q4 (>4.89)	1.05 (0.91‐1.21)	1.04 (0.67‐1.61)	1.10 (0.93‐1.31)	0.92 (0.64‐1.34)	1.00 (0.89‐1.14)	1.19 (0.88‐1.60)	1.01 (0.87‐1.18)	0.70 (0.41‐1.21)
*P*_trend_	.56	.3	.65	.22	.82	.41	.94	.31

Abbreviations: BCC, basal‐cell carcinoma; CI, confidence intervals; EPIC, European Prospective Investigation into Cancer and Nutrition; HR, hazard ratio; SCC, squamous‐cell carcinoma.

^a^ Stratified by age at recruitment, sex, and study center, and adjusted for educational level (none, primary school, technical/professional school, secondary school, university or higher degree), body mass index (BMI; <25, 25‐29, or ≥30 kg/m^2^), smoking (never, former, and current), physical activity level (metabolic equivalent of task [MET] hour/week), total energy intake (continuous), and other alcoholic beverages (quartiles).

^b^ Quartiles for types of alcoholic beverages including beer, wine, liquors, and spirits were calculated separately for baseline and lifetime alcohol consumption among consumers only.

^c^ Analysis of lifetime alcohol intake was based on 363 310 EPIC participants after exclusion of participants from Norway, Sweden, Naples, and Bilthoven—for whom data on lifetime alcohol was missing.

In women, baseline intake of spirits/liquors was positively and linearly associated with a higher skin cancer risk (*P*_trend_ = .002), particularly BCC, although associations were small and not statistically significant in individual quartiles (Table 4). However, average lifetime intake of spirits/liquors was not associated with skin cancer risk (HR = 1.03, 95% CI = 0.94‐1.13, *P*_trend_ = .12). Baseline and lifetime wine intakes were positively associated with skin cancer risk (HR = 1.08, 95% CI = 1.00‐1.16, *P*_trend_ = .09 and HR = 1.09, 95% CI = 1.01‐1.17, *P*_trend_ = .17, respectively), particularly with BCC risk, although again with no significant linear trend. Baseline and lifetime beer intakes were not associated with skin cancer risk in women.

### 3.3 Alcohol intake at different ages

When considering alcohol intake at different ages, in men, the positive linear associations between alcohol intake and skin cancer risk appeared stronger for intake of alcohol at ages 40 and 50 (HR = 1.22, 95% CI = 1.08‐1.38, *P*_trend_ = .0006 and HR = 1.26, 95% CI = 1.09‐1.46, *P*_trend_ = .006, respectively) than for intake at ages 20 and 30 (HR = 1.05, 95% CI = 0.97‐1.14, *P*_trend_ = .02 and HR = 1.09, 95% CI = 0.99‐1.19, *P*_trend_ = .007), although with no significant heterogeneity (*P*_heterogeneity_ = .67) (Table S1).

In contrast, in women, there were small positive but significant linear associations between alcohol intake at different ages and overall skin cancer risk (.0002 ≤ *P*_trend_ ≤ .03). With regards to skin cancer types, melanoma risk was significantly associated with alcohol intake at age 20 (HR = 1.35, 95% CI = 1.07‐1.70, *P*_trend_ = .0001), whereas BCC risk was associated with intakes at ages 40 and 50 years (HR = 1.10, 95% CI = 1.00‐1.22, *P*_trend_ = .008 and HR = 1.14, 95% CI = 1.01‐1.28, *P*_trend_ = .0008, respectively). However, no heterogeneity was found across cancer types (.32 < *P*_heterogeneity_ < .59), and we observed no association with SCC risk across ages of intake.

### 3.4 Subgroup analyses

Stratified analyses by country showed no statistically significant association between baseline alcohol intake and skin cancer risk in all countries with no heterogeneity detected across countries (all *P*_heterogeneity_ ≥ .32). There was no heterogeneity in estimates across countries for separate outcomes (all *P*_heterogeneity_ ≥ .30) (Table S2).

In stratified analyses according to skin cancer site, the positive association between baseline alcohol intake and melanoma risk was stronger for trunk tumors (HR = 1.47, 95% CI = 1.07‐2.03, *P*_trend_ = .02) compared to those of the head, neck or extremities (HR = 0.99, 95% CI = 0.75‐1.32, *P*_trend_ = .93) in men, with statistically significant heterogeneity across tumor sites (*P*_heterogeneity_ = .04) (Table S3). We found no heterogeneity across body sites in women (*P*_heterogeneity_ ≥ .22).

In competing‐risk analyses for histologic type, associations were stronger for superficial spreading melanoma (HR = 1.47, 95% CI = 1.04‐2.07, *P*_trend_ = .004) in men compared to those of other histologic type (*P*_heterogeneity_ = .05). There was no evidence for heterogeneity in findings in women regarding subtype‐specific analyses (*P*_heterogeneity_ ≥ .47) (Table S4).

We found no evidence of effect modification by hours of recreational sun exposure during outdoor physical activity in summer (data not shown).

## 4 DISCUSSION

In this large prospective study with 14 037 incident skin cancer cases developed over a median follow‐up of 15 years, we found that alcohol intake at baseline was linearly associated with an increased risk of skin cancer, particularly with SCC and BCC. Average alcohol intakes during adulthood were also associated with skin cancer risk, particularly with SCC and BCC in men and with BCC in women. Separate analyses by type of alcoholic beverages showed that intakes of liquor/spirits were positively associated with melanoma and BCC risks in men, while wine intake was associated with risks of BCC and SCC. In women, intakes of wine were associated with a higher risk of BCC. Beer intake was not associated with skin cancer risk in both sexes.

With regards to melanoma, most previous cohort studies have found a positive association between alcohol consumption and melanoma risk.^14^, ^15^, ^16^, ^17^, ^18^ Kubo and colleagues reported a positive relationship in postmenopausal women from the Women's Health Initiative (WHI) after an average follow‐up of 10.2 years^15^: women who consumed white wine and liquors had 52% and 65% higher risks of melanoma compared to nondrinkers, respectively. Two prospective cohort studies investigated the association between alcohol intake and skin cancer risk: the Nurses' Health Studies in women (NHS I, 1984‐2012 and NHS II, 1991‐2011) and the Health Professionals Follow‐Up Study in men (HPFS, 1986‐2012).^18^ Research in these cohorts reported that higher intake of alcohol was associated with melanoma risk, with a 14% increased risk per drink per day. Similar to the results from the WHI, participants who consumed white wine (but not red wine) over 5 times per week had 42% higher risks of melanoma compared to nondrinkers after adjustment for known skin cancer risk factors and other alcoholic beverages. Consistently, our findings suggested that alcohol intake at baseline was positively and linearly associated with skin cancer risk, both in men and women. We also found that average lifetime intakes of liquors/spirits were the most strongly associated with melanoma risk, with the highest intakes in this population (>3.08 g/day) associated with a 47% increased melanoma risk compared to the lowest intake (0‐0.13 g/day). While information on intake of different types of wine was not available in EPIC, overall intakes of wine were not significantly associated with melanoma risk. A case‐control study and a pooled analysis of eight case‐control studies reported a positive association between alcohol intake and melanoma risk,^11^, ^27^ while most case‐control studies reported no association between total alcohol intake^19^ or intake of different types of alcoholic beverages^19^, ^27^ and melanoma in either men or women.

Our stratified analyses by skin cancer sites suggested that the positive association between alcohol intake and melanoma risk were stronger for tumors occurring on the trunk compared to those of the head, neck or extremities in men. These findings corroborate those previously found in the NHS and HPFS cohorts.^18^ Similarly, a pooled analysis of eight case‐control studies additionally found a stronger association between alcohol intake and melanoma risk in tumors of the trunk compared to tumors at other body sites.^11^ In addition, our stratified analyses by histologic type showed that baseline alcohol intake was mostly associated with superficial spreading melanoma in men. According to the divergent pathway model for melanoma, trunk melanomas or superficial spreading melanomas are associated with nevus propensity and genetic susceptibility, while melanomas occurring on the head/neck or of the lentigo maligna type are associated with chronic sun exposure.^25^ Our observations may lend support to the hypothesis that the relationship between alcohol intake and melanoma could differ by tumor site and type, and possibly also suggest that alcohol could affect melanoma through pathways involving genetic susceptibility rather than pathways involving chronic sun exposure. Further studies with detailed information on confounders such as sun exposure behaviors are needed to investigate potential gene‐environment interactions between alcohol and UV exposure on melanoma risk.

Regarding KCs, most case‐control studies have found no association between alcohol consumption and the risks of BCC or SCC.^28^, ^29^, ^30^, ^31^, ^32^ However, a large case‐control study involving 57 121 BCC cases and 57 121 controls found a modest positive association between alcohol intake and BCC risk.^33^ Evidence from cohort studies reported an increased risk of KCs associated with alcohol intake.^15^, ^16^, ^17^, ^34^, ^35^, ^36^ In particular, Kubo and colleagues found a positive association between higher levels of current alcohol intake and KC risk in the WHI cohort, suggesting a 23% increase in KC risk for those reporting 7+ drinks per week compared to nondrinkers.^15^ They observed that increasing lifetime alcohol intake was also positively associated with KC risk. The greater risk of KC was observed in participants with a preference for white wine and for liquors vs alcohol nondrinkers. Similarly, findings from the NHS and HPFS cohorts showed that alcohol intake was associated with BCC and SCC risks, and that the positive associations appeared stronger with white wine and liquor intakes.^16^, ^17^ Moreover, white wine and liquor consumption were also associated with higher risk of BCC in a Danish cohort of men and women.^35^ In line with these results, we found that alcohol intake was positively and linearly associated with the risks of BCC and SCC in men and with BCC in women. Specifically, consumption of liquors/spirits and wine was associated with a higher BCC risk in our study, although we were not able to investigate association between risk of skin cancer and different types of wine (white vs red wine).

The most recent WCRF/AICR report concluded that limited but suggestive evidence existed on the relationship between alcoholic drinking and risks of melanoma and BCC, in agreement with findings from prospective studies suggesting that alcohol consumption increased the risk of skin cancer, consistently with findings from several meta‐analyses.^20^, ^21^, ^22^

Experimental studies have shown that alcohol by itself is not carcinogenic, but ethanol metabolism into acetaldehyde by alcohol dehydrogenase may play a major role in carcinogenesis, for example by inhibiting the DNA repair system.^3^ Ethanol metabolism also induces the production of reactive oxygen species, which cause DNA damage and contribute to the formation of mutagenic adducts,^3^, ^37^ activation of prostaglandin synthesis pathways, and lead to skin carcinogenesis.^8^ Specifically for skin cancer, alcohol metabolites were suggested to have photosensitizing effects,^8^ which can enhance cellular damage and the immunosuppressive effects.^38^ Previous studies reported that alcohol consumers were more likely to report larger numbers of sunburns compared to nonconsumers.^39^, ^40^ Recent results from the French E3N cohort showed that alcohol drinkers were more likely to use sunbeds than nondrinkers.^41^ As a result, participants reporting higher alcohol intake may have riskier behaviors toward UV exposure including a more frequent use of sunbeds or have higher skin sensitivity making them more susceptible to skin cancer. Unfortunately, data on established risk factors for skin cancer were not available in the EPIC cohort, which prevented us from adjusting for sun exposure. However, adjustment for sun exposure in previous studies did not make a substantial difference on the results^16^, ^18^, ^27^ and we found similar results after adjusting for hours of recreational sun exposure during outdoor physical activity in summer. The increased risk of skin cancer associated with wine and liquor/spirits intakes could be explained by higher amounts of acetaldehyde present in wine and liquor compared to beer, as previously reported.^37^, ^42^, ^43^ In addition, we found that nondrinkers among men had a 20% higher risk of BCC compared to those who reported average lower intake of liquor and spirit during lifetime (>0‐0.13 g/day). Nondrinkers among women had a 32% higher risk of melanoma compared to those who reported average lower intake of wine during lifetime (>0‐0.14 g/day). We could hypothesize that nonalcoholic drinkers may have unhealthier sun exposure behaviors or tend to have pigmentary traits which lead them to higher skin cancer risk compared to those who had moderate alcohol intake. However, we lacked data on sun exposure and other skin cancer risk factors, and ideally, future studies should investigate this hypothesis.

Strengths of our study include the large sample size of EPIC and availability of data on baseline alcohol intakes in 10 European countries and lifetime alcohol intakes in 6 European countries, spanning a wide diversity of alcohol intakes across Europe; the prospective design, particularly the long duration of follow‐up; and information on melanoma type and skin cancer site. In addition, skin cancer cases were confirmed using a combination of different methods, and dietary and lifestyle questionnaires were assessed using validated dietary questionnaires in all centers. This is the first and largest prospective study to date that investigated the relationship between alcohol consumption at different ages on skin cancer risk. The study also has limitations. First, the lack of information on skin cancer risk factors, such as sun exposure, pigmentary traits, and family history of skin cancer.^44^, ^45^ Although we used hours of recreational physical activity in summer as a proxy for time spent outdoors, information on sun exposure behaviors was not available, likely leading to potential for residual confounding. Exposure misclassification of study participants in estimating their alcohol intake cannot be excluded, particularly at early ages during their adulthood. In addition, while melanoma cases are generally accurately recorded, KCs are often not tracked by cancer registries; thus, the reported incidence of KC is likely underestimated in our study.

In conclusion, in this large European prospective study, baseline and lifetime alcohol intakes were positively and linearly associated with the risk of skin cancer, particularly with SCC and BCC. While intake of liquors/spirits was associated with melanoma risk in men, intake of wine was associated with risks of both BCC and SCC in men and only BCC in women. If replicated, these findings may have important public health implications in the primary prevention of skin cancer.

## AUTHOR CONTRIBUTIONS

**Yahya Mahamat‐Saleh** performed the statistical analyses. **Yahya Mahamat‐Saleh** drafted the manuscript. **Marina Kvaskoff** was responsible for supervision, conceptualization, and methodology. All authors contributed to the acquisition and interpretation of data and critically revised the manuscript for important intellectual content. All authors have read and approved the final manuscript for publication. The work reported in the study has been performed by the authors, unless clearly specified in the text.

## FUNDING INFORMATION

Yahya Mahamat‐Saleh was supported by the French National Cancer Institute (INCa). The coordination of EPIC is financially supported by the European Commission (DG‐SANCO); and the International Agency for Research on Cancer. The national cohorts are supported by Danish Cancer Society (Denmark); the French National Institute of Health and Medical Research (Inserm), the Mutuelle Générale de l'Education Nationale, the Gustave Roussy Institute, and the French League against Cancer (France); Deutsche Krebshilfe, Deutsches Krebsforschungszentrum (DKFZ); and Federal Ministry of Education and Research (Germany); Stavros Niarchos Foundation; the Hellenic Health Foundation; and Ministry of Health and Social Solidarity (Greece); Italian Association for Research on Cancer (AIRC); National Research Council; and AIRE‐ONLUS Ragusa, AVIS Ragusa, Sicilian Government (Italy); Dutch Ministry of Public Health, Welfare and Sports (VWS); Netherlands Cancer Registry (NKR); LK Research Funds; Dutch Prevention Funds; Dutch ZON (Zorg Onderzoek Nederland); World Cancer Research Fund (WCRF); the National Institute for Public Health and the Environment (RIVM) and Statistics Netherlands (the Netherlands); European Research Council (ERC) (grant number ERC‐2009‐AdG 232997) and Nordforsk; and Nordic Center of Excellence Programme on Food, Nutrition and Health (Norway); Health Research Fund (FIS); Regional Governments of Andalucía, Asturias, Basque Country, Murcia (No. 6236) and Navarra; and ISCIII RETIC (RD06/0020) and the Catalan Institute of Oncology. (Spain); Swedish Cancer Society; Swedish Scientific Council; and Regional Government of Skåne and Västerbotten (Sweden); Cancer Research UK (14136 to EPIC‐Norfolk; C570/A16491 and C8221/A19170 for EPIC‐Oxford); Medical Research Council (1000143 to EPIC‐Norfolk, MR/M012190/1 to EPIC‐Oxford); Stroke Association; British Heart Foundation; Department of Health; Food Standards Agency; and Wellcome Trust (UK).

## CONFLICT OF INTEREST

The authors have no conflict of interest to disclose.

## ETHICS STATEMENT

The EPIC study was conducted in accordance with the Declaration of Helsinki. The study was approved by the local ethical committees in participating countries and the IARC ethical committee. All participants provided written informed consent for data collection and storage, as well as individual follow‐up before study entry. The EPIC Steering Committee approved our study in accordance with EPIC rules: https://epic.iarc.fr/access/index.php

## Supporting information


**Table S1** Supporting information

## Data Availability

The data that support the findings of our study are available from the corresponding author upon reasonable request. Please follow the instructions at http://epic.iarc.fr/access/index.php
