# Supplementary material for: Baseline and lifetime alcohol consumption and risk of skin cancer in the European Prospective Investigation into Cancer and Nutrition cohort (EPIC)
Source: Int J Cancer. 2022 Aug 30;152(3):348–62. doi: 10.1002/ijc.34253 (PMC10087036; doi:10.1002/ijc.34253)
Supplement: Supplementary file 1 — Table S1 Supporting information [file IJC-152-348-s001.pdf]

Baseline and lifetime alcohol consumption and risk of skin cancer in the European Prospective Investigation into Cancer and nutrition cohort (EPIC)

Yahya Mahamat-Saleh, Marie Al-Rahmoun, Gianluca Severi G, Reza Ghiasvand, Marit B. Veierod, Saverio Caini, Domenico Palli, Edoardo Botteri, Carlotta Sacerdote, Fulvio Ricceri, Marko Lukic, Maria J. Sánchez, Valeria Pala, Rosario Tumino, Paolo Chiodini, Pilar Amiano, Sandra Colorado-Yohar, María-Dolores Chirlaque, Eva Ardanaz, Catalina Bonet, Verena Katzke, Rudolf Kaaks, Matthias B. Schulze, Kim Overvad, Christina C. Dahm, Christian S. Antoniussen, Anne Tjønneland, Cecilie Kyrø, Bas Bueno-de-Mesquita, Jonas Manjer, Malin Jansson, Anders Esberg, Nagisa Mori, Pietro Ferrari, Elisabete Weiderpass, Marie-Christine Boutron-Ruault, Marina Kvaskoff

Table of contents

1. **Supplementary Table S1:** Hazard ratios (HRs) and 95% confidence intervals (CIs) for intake of alcohol at different ages and risk of skin cancer, by sex, EPIC cohort
2. **Supplementary Table S2:** Hazard ratios (HRs) and 95% confidence intervals (CIs) for intake of baseline and lifetime alcohol and risk of skin cancer (per 12 g per day among drinkers) according to sex, and country, EPIC cohort
3. **Supplementary Table S3:** Hazard ratios (HRs) and 95% confidence intervals (CIs) for baseline and lifetime intake of alcohol and risk of skin cancer according to sex and tumor site, EPIC cohort
4. **Supplementary Table S4:** Hazard ratios (HRs) and 95% confidence intervals (CIs) for intake of baseline and lifetime alcohol and risk of melanoma according to histologic type, and sex, EPIC cohort

Supplementary Table S1: Hazard ratios (HRs) and 95% confidence intervals (CIs) for intake of alcohol at different ages and risk of skin cancer, by sex, EPIC cohort

	Men				Women			
	**Skin cancer**	**Melanoma**	**BCC**	**SCC**	**Skin cancer**	**Melanoma**	**BCC**	**SCC**
	HR (95% CI)[1]	HR (95% CI)[1]	HR (95% CI)[1]	HR (95% CI)[1]	HR (95% CI)[1]	HR (95% CI)[1]	HR (95% CI)[1]	HR (95% CI)[1]
**Alcohol consumption at age 20 years (g/d)**[2]								
Non-drinkers	0.94 (0.84-1.05)	1.08 (0.79-1.49)	0.92 (0.81-1.05)	0.97 (0.72-1.30)	0.92 (0.86-0.97)	0.83 (0.71-0.97)	0.94 (0.88-1.01)	0.88 (0.74-1.05)
>0-4.9	1.00 (reference)	1.00 (reference)	1.00 (reference)	1.00 (reference)	1.00 (reference)	1.00 (reference)	1.00 (reference)	1.00 (reference)
5-14.9	1.07 (0.99-1.16)	1.01 (0.80-1.27)	1.08 (0.99-1.18)	1.15 (0.92-1.44)	1.01 (0.94-1.09)	1.02 (0.85-1.22)	1.05 (0.97-1.14)	0.89 (0.72-1.11)
>15	1.05 (0.97-1.14)	0.99 (0.78-1.26)	1.08 (0.98-1.19)	0.99 (0.77-1.27)	1.06 (0.96-1.18)	1.35 (1.07-1.70)	1.02 (0.90-1.16)	1.03 (0.71-1.50)
P-trend	0.02	0.67	0.007	0.74	0.0002	<.0001	0.01	0.48
**Alcohol consumption at age 30 years (g/d)**[2]								
Non-drinkers	0.94 (0.82-1.07)	0.89 (0.58-1.35)	0.93 (0.79-1.09)	1.00 (0.71-1.42)	0.92 (0.86-0.99)	0.89 (0.74-1.06)	0.94 (0.86-1.01)	0.90 (0.71-1.11)
>0-4.9	1.00 (reference)	1.00 (reference)	1.00 (reference)	1.00 (reference)	1.00 (reference)	1.00 (reference)	1.00 (reference)	1.00 (reference)
5-14.9	1.04 (0.95-1.14)	0.98 (0.75-1.28)	1.03 (0.92-1.14)	1.10 (0.86-1.43)	1.02 (0.96-1.08)	1.07 (0.90-1.26)	1.03 (0.95-1.11)	1.03 (0.83-1.28)
>15	1.09 (0.99-1.19)	1.03 (0.79-1.34)	1.08 (0.97-1.20)	1.09 (0.84-1.41)	1.00 (0.92-1.09)	1.01 (0.81-1.25)	1.03 (0.93-1.14)	0.88 (0.64-1.21)
P-trend	0.007	0.48	0.03	0.49	0.008	0.08	0.02	0.56
**Alcohol consumption at age 40 years (g/d)**[2]								
Non-drinkers	1.02 (0.83-1.26)	1.10 (0.62-1.93)	0.97 (0.76-1.23)	1.71 (0.84-3.45)	0.95 (0.87-1.02)	0.95 (0.79-1.16)	0.95 (0.86-1.05)	0.88 (0.68-1.15)
>0-4.9	1.00 (reference)	1.00 (reference)	1.00 (reference)	1.00 (reference)	1.00 (reference)	1.00 (reference)	1.00 (reference)	1.00 (reference)
5-14.9	1.14 (1.00-1.30)	0.98 (0.67-1.42)	1.12 (0.96-1.30)	1.72 (1.02-2.91)	1.01 (0.94-1.08)	0.95 (0.80-1.15)	1.02 (0.94-1.11)	1.07 (0.86-1.34)
>15	1.22 (1.08-1.38)	1.17 (0.83-1.65)	1.16 (1.01-1.34)	2.04 (1.23-3.36)	1.05 (0.96-1.14)	0.96 (0.77-1.19)	1.10 (1.00-1.22)	0.95 (0.71-1.26)
P-trend	0.0006	0.25	0.01	0.02	0.03	0.9	0.008	0.43
**Alcohol consumption at age 50 years (g/d)**[2]								
Non-drinkers	1.14 (0.89-1.47)	0.76 (0.48-1.19)	0.95 (0.71-1.28)	1.35 (0.50-3.59)	0.87 (0.76-1.00)	0.76 (0.48-1.19)	0.89 (0.76-1.04)	0.82 (0.46-1.44)
>0-4.9	1.00 (reference)	1.00 (reference)	1.00 (reference)	1.00 (reference)	1.00 (reference)	1.00 (reference)	1.00 (reference)	1.00 (reference)
5-14.9	1.18 (1.01-1.38)	0.93 (0.66-1.30)	1.10 (0.93-1.31)	1.67 (0.90-3.11)	1.06 (0.96-1.18)	0.93 (0.66-1.30)	1.08 (0.96-1.20)	1.09 (0.72-1.65)
>15	1.26 (1.09-1.46)	0.96 (0.66-1.40)	1.16 (0.99-1.37)	1.83 (1.01-3.33)	1.09 (0.98-1.22)	0.96 (0.66-1.40)	1.14 (1.01-1.28)	0.85 (0.52-1.40)
P-trend	0.006	0.51	0.02	0.07	0.001	0.51	0.0008	0.90

Abbreviations: BCC: basal-cell carcinoma, CI: confidence intervals, EPIC: European Prospective Investigation into Cancer and Nutrition, HR: hazard ratio: SCC: squamous-cell carcinoma

[1]Stratified by age at recruitment, sex, and study center, and adjusted for educational level (none, primary school, technical/professional school, secondary school, university or higher degree), body mass index (BMI; <25, 25–29, or ≥30 kg/m^2), smoking (never, former, and current), physical activity level (metabolic equivalent of task [MET] hour/week), and total energy intake (continuous)

[2]Analysis was based on 363,310 EPIC participants after exclusion of participants from Norway, Sweden, Naples, and Bilthoven – for whom data on lifetime alcohol was missing.

Supplementary Table S2: Hazard ratios (HRs) and 95% confidence intervals (CIs) for intake of baseline and lifetime alcohol and risk of skin cancer (per 12 g per day among drinkers) according to sex, and country, EPIC cohort

		Men		Women	
		Baseline alcohol Intake	Average lifetime intake of alcohol	Baseline alcohol Intake	Average lifetime intake of alcohol
		HR[1] (95% CI)	HR[1] (95% CI)	HR[1] (95% CI)	HR[1] (95% CI)
Denmark					
	Skin cancer	0.98 (0.97-1.00)	1.13 (0.71-1.79)	1.04 (1.00-1.07)	2.56 (1.51-4.34)
	Melanoma	0.97 (0.91-1.03)	0.55 (0.12-2.47)	0.97 (0.87-1.07)	0.47 (0.07-3.26)
	BCC	0.99 (0.96-1.01)	1.23 (0.74-2.07)	1.04 (1.00-1.07)	3.14 (1.77-5.58)
	SCC	1.00 (0.94-1.07)	1.19 (0.24-5.80)	1.01 (0.89-1.14)	2.14 (0.21-21.45)
United Kingdom					
	Skin cancer	0.99 (0.96-1.03)	1.80 (0.99-3.28)	1.04 (0.99-1.09)	1.42 (0.66-3.05)
	Melanoma	0.97 (0.87-1.10)	2.25 (0.36-14.25)	0.99 (0.87-1.14)	2.07 (0.44-9.68)
	BCC	0.98 (0.93-1.02)	1.45 (0.70-2.99)	1.05 (0.98-1.11)	1.42 (0.66-3.05)
	SCC	1.05 (0.97-1.14)	4.32 (1.10-17.04)	1.06 (0.92-1.21)	1.37 (0.26-7.33)
The Netherlands					
	Skin cancer	1.06 (0.97-1.17)		0.98 (0.90-1.07)	0.49 (0.13-1.82)
	Melanoma	1.00 (0.85-1.18)		0.99 (0.87-1.13)	0.76 (0.01-7.25)
	BCC	_		0.93 (0.65-1.33)	1.15 (0.01-156.90)
	SCC	1.10 (0.96-1.25)		0.99 (0.88-1.12)	0.44 (0.07-2.68)
Germany					
	Skin cancer	1.01 (0.97-1.05)	1.75 (0.64-4.82)	1.02 (0.95-1.10)	1.00 (0.25-3.94)
	Melanoma	1.04 (0.96-1.13)	1.64 (0.19-14.10)	0.91 (0.75-1.11)	0.41 (0.02-7.72)
	BCC	1.00 (0.95-1.05)	2.32 (0.67-8.07)	1.06 (0.97-1.15)	1.35 (0.27-6.72)
	SCC	1.02 (0.87-1.18)	0.25 (0.01-7.76)	0.70 (0.33-1.48)	_
France					
	Skin cancer	_		0.99 (0.95-1.03)	0.81 (0.47-1.40)
	Melanoma	_		1.00 (0.92-1.09)	1.03 (0.31-3.46)
	BCC	_		0.97 (0.91-1.02)	0.75 (0.34-1.64)
	SCC	_		1.05 (0.92-1.19)	0.44 (0.10-3.39)
Spain					
	Skin cancer	0.99 (0.95-1.03)	1.28 (0.48-3.40)	0.89 (0.74-1.07)	0.49 (0.15-1.61)
	Melanoma	1.02 (0.92-1.13)	0.62 (0.05-8.08)	0.88 (0.58-1.34)	0.18 (0.01-3.13)
	BCC	0.97 (0.92-1.03)	1.13 (0.35-3.68)	0.86 (0.68-1.09)	0.40 (0.1-1.66)
	SCC	0.98 (0.89-1.09)	4.02 (0.29-56.24)	1.15 (0.70-1.90)	_
Italy					
	Skin cancer	1.00 (0.95-1.06)	0.85 (0.31-2.36)	1.00 (0.93-1.08)	1.04 (0.39-2.78)
	Melanoma	1.00 (0.88-1.14)	1.33 (0.11-16.13)	1.09 (0.93-1.28)	3.58 (0.32-39.56)
	BCC	0.98 (0.92-1.04)	0.53 (0.16-1.72)	0.98 (0.90-1.08)	0.85 (0.27-2.72)
	SCC	1.14 (0.92-1.41)	3.33 (0.04-15.25)	0.76 (0.47-1.23)	1.26 (0.01-189.81)
Sweden					
	Skin cancer	_	_	0.99 (0.92-1.07)	_
	Melanoma	_	_	0.96 (0.86-1.09)	_
	BCC	_	_	0.14 (0.01-1.66)	_
	SCC	_	_	1.02 (0.92-1.13)	_
Norway					
	Skin cancer	_	_	1.51 (1.06-2.15)	_
	Melanoma	_	_	1.31 (0.84-2.03)	_
	BCC	_	_	_	_
	SCC	_	_	1.64 (0.81-3.31)	_

Abbreviations: BCC: basal-cell carcinoma, CI: confidence intervals, EPIC: European Prospective Investigation into Cancer and Nutrition, HR: hazard ratio: SCC: squamous-cell carcinoma

[1]Stratified by age at recruitment, sex, and study center, and adjusted for educational level (none, primary school, technical/professional school, secondary school, university or higher degree), body mass index (BMI; <25, 25–29, or ≥30

kg/m2), smoking (never, former, and current), physical activity level (metabolic equivalent of task [MET] hour/week), and total energy intake (continuous)

Supplementary Table S3: Hazard ratios (HRs) and 95% confidence intervals (CIs) for **baseline and lifetime intake of alcohol** and risk of skin cancer according to sex and tumor site, EPIC cohort

	**Skin cancer**		**Melanoma**		**BCC**		**SCC**	
	Head, neck and extremities	Trunk	Head, neck and extremities	Trunk	Head, neck and extremities	Trunk	Head, neck and extremities	Trunk
	HR[1] (95% CI)	HR[1] (95% CI)	HR[1] (95% CI)	HR[1] (95% CI)	HR[1] (95% CI)	HR[1] (95% CI)	HR[1] (95% CI)	HR[1] (95% CI)
**Men**								
**Baseline total alcohol intake (g/d)**								
Non-drinkers	0.95(0.80-1.12)	1.19 (0.87-1.64)	0.95 (0.60-1.51)	1.05 (0.60-1.83)	0.97 (0.78-1.20)	1.22 (0.79-1.90)	1.05 (0.75-1.48)	1.40 (0.52-3.79)
>0-4.9	1.00 (reference)	1.00 (reference)	1.00 (reference)	1.00 (reference)	1.00 (reference)	1.00 (reference)	1.00 (reference)	1.00 (reference)
5-14.9	1.11 (1.01-1.24)	1.29 (1.07-1.57)	0.99 (0.75-1.31)	1.05 (0.60-1.83)	1.12 (0.98-1.28)	1.27 (0.98-1.64)	1.35 (1.08-1.70)	1.36 (0.69-2.69)
>15	1.15 (1.04-1.28)	1.30 (1.08-1.57)	0.99 (0.75-1.32)	1.47 (1.07-2.03)	1.14 (1.00-1.30)	1.14 (0.89-1.47)	1.40 (1.11-1.76)	1.67 (0.85-3.28)
P-trend	0.001	0.04	0.93	0.02	0.03	0.77	0.004	0.23
**Average lifetime alcohol intake (g/d)**[2]								
Non-drinkers	0.98 (0.70-1.38)	0.96 (0.47-1.98)	1.40 (0.49-3.98)	0.93 (0.22-3.96)	1.01 (0.68-1.51)	0.94 (0.38-2.36)	0.73 (0.29-1.82)	_
>0-4.9	1.00 (reference)	1.00 (reference)	1.00 (reference)	1.00 (reference)	1.00 (reference)	1.00 (reference)	1.00 (reference)	1.00 (reference)
5-14.9	1.13 (1.01-1.27)	1.19 (0.96-1.48)	1.03 (0.70-1.51)	1.04 (0.68-1.59)	1.16 (1.00-1.33)	1.30 (0.99-1.70)	1.12 (0.85-1.48)	0.91 (0.39-2.12)
>15	1.15 (1.02-1.30)	1.17 (0.95-1.45)	0.97 (0.66-1.43)	1.15 (0.77-1.74)	1.15 (1.00-1.33)	1.21 (0.92-1.58)	1.27 (0.96-1.69)	0.98 (0.42-2.30)
P-trend	0.03	0.23	0.64	0.39	0.10	0.35	0.05	0.81
**Women**								
**Baseline total alcohol intake (g/d)**								
Non-drinkers	1.08 (0.99-1.18)	0.82 (0.67-1.01)	1.13 (0.95-1.36)	0.87 (0.63-1.22)	1.08 (0.95-1.22)	0.86 (0.65-1.14)	1.02 (0.84-1.24)	0.57 (0.27-1.19)
>0-4.9	1.00 (reference)	1.00 (reference)	1.00 (reference)	1.00 (reference)	1.00 (reference)	1.00 (reference)	1.00 (reference)	1.00 (reference)
5-14.9	1.09 (1.02-1.17)	0.98 (0.86-1.12)	1.02 (0.89-1.18)	0.87 (0.67-1.12)	1.12 (1.03-1.23)	0.99 (0.83-1.18)	1.09 (0.93-1.29)	1.13 (0.71-1.81)
>15	1.03 (0.95-1.12)	0.93 (0.79-1.09)	0.95 (0.79-1.13)	0.78 (0.56-1.08)	1.05 (0.95-1.16)	0.94 (0.77-1.14)	1.05 (0.86-1.29)	1.31 (0.75-2.28)
P-trend	0.75	0.72	0.20	0.30	0.44	0.95	0.50	0.05
**Average lifetime alcohol intake (g/d)**[2]								
Non-drinkers	1.12 (1.00-1.26)	0.89 (0.67-1.17)	1.21 (0.94-1.57)	0.99 (0.58-1.71)	1.12 (0.97-1.29)	0.87 (0.62-1.24)	0.93 (0.69-1.26)	0.78 (0.23-2.67)
>0-4.9	1.00 (reference)	1.00 (reference)	1.00 (reference)	1.00 (reference)	1.00 (reference)	1.00 (reference)	1.00 (reference)	1.00 (reference)
5-14.9	1.02 (0.96-1.09)	1.08 (0.94-1.24)	1.03 (0.88-1.21)	1.04 (0.77-1.40)	1.03 (0.95-1.12)	1.05 (0.89-1.24)	1.01 (0.84-1.20)	1.30 (0.74-2.27)
>15	1.03 (0.93-1.13)	1.09 (0.91-1.31)	1.08 (0.87-1.34)	1.28 (0.88-1.86)	1.05 (0.93-1.17)	0.99 (0.79-1.25)	0.90 (0.69-1.19)	1.46 (0.70-3.02)
P-trend	0.72	0.13	0.93	0.28	0.97	0.60	0.83	0.20

Abbreviations: BCC: basal-cell carcinoma, CI: confidence intervals, EPIC: European Prospective Investigation into Cancer and Nutrition, HR: hazard ratio: SCC: squamous-cell carcinoma

[1]Stratified by age at recruitment, sex, and study center, and adjusted for educational level (none, primary school, technical/professional school, secondary school, university or higher degree), body mass index (BMI; <25, 25–29, or ≥30 kg/m^2), smoking (never, former, and current), physical activity level (metabolic equivalent of task [MET] hour/week), and total energy intake (continuous)

[2]Analysis of lifetime alcohol intake was based on 363,310 EPIC participants after exclusion of participants from Norway, Sweden, Naples, and Bilthoven – for whom data on lifetime alcohol was missing.

Supplementary Table S4: Hazard ratios (HRs) and 95% confidence intervals (CIs) for intake of **baseline and lifetime alcohol** and risk of melanoma according to histologic type, and sex, EPIC cohort

Histologic type of melanoma	Lentigo maligna	Superficial spreading	Acro lentiginous	Nodular	Other[2]	
	HR[1] (95% CI)	HR[1] (95% CI)	HR[1] (95% CI)	HR[1] (95% CI)	HR[1] (95% CI)	$P_{heterogeneity}$
**Men**						
**Baseline total alcohol intake (g/d)**						
Cases	62	327	9	79	335	
Non-drinkers	1.17 (0.43-3.17)	0.62 (0.29-1.33)	1.65 (0.13-20.76)	1.00 (0.39-2.59)	1.34 (0.84-2.14)	
>0-4.9	1.00 (reference)	1.00 (reference)	1.00 (reference)	1.00 (reference)	1.00 (reference)	
5-14.9	1.17 (0.56-2.45)	1.36 (0.95-1.96)	1.40 (0.23-8.70)	0.66 (0.35-1.26)	0.99 (0.73-1.35)	
>15	1.37 (0.66-2.86)	1.47 (1.04-2.07)	0.80 (0.12-5.43)	0.64 (0.35-1.17)	1.09 (0.80-1.50)	0.05
P-trend	0.50	0.004	0.63	0.13	0.82	
**Average lifetime alcohol intake (g/d)**[3]						
Cases	51	263	7	56	178	
Non-drinkers	2.51 (0.50-12.70)	0.50 (0.07-3.71)	_	2.51 (0.50-12.70)	2.91 (1.09-7.79)	
>0-4.9	1.00 (reference)	1.00 (reference)	1.00 (reference)	1.00 (reference)	1.00 (reference)	
5-14.9	1.45 (0.59-3.53)	1.14 (0.75-1.74)	0.57 (0.03-10.23)	1.45 (0.59-3.53)	0.78 (0.48-1.27)	
>15	1.29 (0.50-3.31)	1.16 (0.77-1.75)	1.89 (0.17-20.76)	1.29 (0.50-3.31)	1.07 (0.68-1.68)	0.47
P-trend	0.97	0.40	0.37	0.97	0.95	
**Women**						
**Baseline total alcohol intake (g/d)**						
Cases	139	865	24	116	490	
Non-drinkers	1.16 (0.72-1.87)	1.22 (0.99-1.51)	1.44 (0.47-4.38)	1.33 (0.79-2.23)	0.76 (0.55-1.04)	
>0-4.9	1.00 (reference)	1.00 (reference)	1.00 (reference)	1.00 (reference)	1.00 (reference)	
5-14.9	0.84 (0.54-1.30)	1.08 (0.91-1.27)	0.65 (0.21-1.98)	1.03 (0.64-1.64)	0.88 (0.70-1.10)	
>15	0.74 (0.44-1.25)	0.88 (0.72-1.08)	0.69 (0.20-2.42)	0.68 (0.33-1.40)	1.18 (0.90-1.54)	0.63
P-trend	0.10	0.07	0.22	0.16	0.10	
**Average lifetime alcohol intake (g/d)**[3]						
Cases	122	21	661	61	293	
Non-drinkers	1.53 (0.88-2.64)	1.29 (0.97-1.73)	1.86 (0.46-7.53)	0.36 (0.10-1.30)	0.93 (0.54-1.62)	
>0-4.9	1.00 (reference)	1.00 (reference)	1.00 (reference)	1.00 (reference)	1.00 (reference)	
5-14.9	0.84 (0.53-1.31)	0.94 (0.78-1.12)	1.92 (0.70-5.29)	0.85 (0.46-1.57)	1.32 (1.00-1.72)	
>15	1.13 (0.62-2.06)	0.97 (0.76-1.23)	1.10 (0.22-5.58)	1.23 (0.56-2.68)	1.50 (1.06-2.12)	0.51
P-trend	0.31	0.17	0.86	0.31	0.01	

Abbreviations: BCC: basal-cell carcinoma, CI: confidence intervals, EPIC: European Prospective Investigation into Cancer and Nutrition, HR: hazard ratio: SCC: squamous-cell carcinoma

[1]Stratified by age at recruitment, sex, and study center, and adjusted for educational level (none, primary school, technical/professional school, secondary school, university or higher degree), body mass index (BMI; <25, 25–29, or ≥30 kg/m^2), smoking (never, former, and current), physical activity level (metabolic equivalent of task [MET] hour/week), and total energy intake (continuous). N=7 melanoma cases with missing histologic type were excluded for the analyses of skin cancer types.

[2]Others include melanoma that is not specified

[3]Analysis of lifetime alcohol intake was based on 363,310 EPIC participants after exclusion of participants from Norway, Sweden, Naples, and Bilthoven – for whom data on lifetime alcohol was missing.
